# Knowledge about diabetes and its association with adherence to self-care and glycemic control in patients with type 1 diabetes in Southern Brazil

**DOI:** 10.20945/2359-3997000000648

**Published:** 2023-06-19

**Authors:** Luciana Foppa, Betina Nemetz, Rosimeri De Matos, Josiane Schneiders, Gabriela Heiden Telo, Beatriz D. Schaan

**Affiliations:** 1 Hospital de Clínicas de Porto Alegre Porto Alegre RS Brasil Hospital de Clínicas de Porto Alegre (HCPA), Porto Alegre, RS, Brasil; 2 Universidade Federal do Rio Grande do Sul Porto Alegre RS Brasil Programa de Pós-graduação em Endocrinologia, Universidade Federal do Rio Grande do Sul, Porto Alegre, RS, Brasil; 3 Universidade Federal do Rio Grande do Sul Escola de Enfermagem Porto Alegre RS Brasil Escola de Enfermagem, Universidade Federal do Rio Grande do Sul, Porto Alegre, RS, Brasil; 4 Pontifícia Universidade Católica do Rio Grande do Sul Hospital São Lucas Porto Alegre RS Brasil Hospital São Lucas da Pontifícia Universidade Católica do Rio Grande do Sul, Porto Alegre, RS, Brasil

**Keywords:** Type 1 diabetes mellitus, knowledge, self-care, glycemic control, health promotion

## Abstract

**Objective:**

To evaluate the association between knowledge about the disease, adherence to self-care, and glycemic control in people diagnosed with type 1 diabetes mellitus.

**Subjects and methods:**

A cross-sectional study of patients aged over 18 years diagnosed with type 1 diabetes mellitus, treated at an outpatient clinic of a Brazilian university hospital. Participants with other types of diabetes, cognitive impairment, pregnancy, and outpatient discharge were excluded. Data were collected from January to March 2021 (by telephone call), with questions about the participants’ profile, diabetes knowledge questionnaire (DKN-A), and self-care inventory revised (SCI-R) translated into and adapted for Brazilian Portuguese. Data analysis involved chi-square associations, Mann-Whitney U tests, and Poisson regression.

**Results:**

Among 198 adult participants, the mean age was 42 ± 12 years, 53.5% were women, the mean glycated hemoglobin was 8.6 ± 1.6%, 140 (70.8%) had satisfactory knowledge about diabetes, 65 (32.8%) had adherence to self-care, and 46 (23.2%) had adequate glycemic control. We found an association between knowledge and adherence to self-care (p < 0.001). Knowledge was not associated with glycemic control (p = 0.705).

**Conclusion:**

Knowledge about diabetes was associated with greater adherence to self-care in people with type 1 diabetes mellitus, but it did not reflect in better glycemic control.

## INTRODUCTION

Diabetes mellitus is a chronic metabolic condition that affects 16.8 million people in Brazil and worldwide. Currently, Brazil ranks third regarding prevalence of type 1 diabetes mellitus (T1DM) cases worldwide and has an estimated number of 92,300 cases in people under 20 years of age (1). People living with diabetes are at greater risk of developing acute and chronic complications (1, 2). These patients need to perform complex self-care activities to obtain good metabolic control for preventing these outcomes (2).

The constant challenge that diabetes represents to those who live with it is a topic of paramount importance. Many patients have difficulties in adhering to the lifestyle changes necessary to promote effective glycemic control and self-care (3, 4). Disturbances in glycemic control, with hyperglycemic peaks, can sometimes be related to lack of knowledge about the disease and negligence with self-care, compromising the health of people with diabetes (3, 4). Interventions by healthcare providers are often insufficient to ensure the effectiveness of diabetes treatment and to prevent its complications, as they may depend on the individual's knowledge about their disease, as well as care to maintain an adequate lifestyle with diabetes (2, 5). Knowledge works together with motivational factors, driving self-care actions, thus, with a better understanding of the disease, interventions can become more effective and uncomplicated to achieve the goal of glycemic control (4, 6).

Studies conducted in different countries show that patients with type 1 diabetes have low to medium knowledge about the disease (7, 8). Brazilian studies have been carried out in people with type 1 and type 2 diabetes, and involve both knowledge of the disease and its complications. These study reported that participants have low knowledge about the disease (4,5,9).

Important factors to be considered for the adequate disease treatment are: analyzing the level of knowledge about the disease, understanding the extent of diabetes acceptance, establishing new ways of providing guidelines, and confirming the effectiveness of healthcare providers’ actions aimed at people with T1DM. The use of validated instruments makes it possible to standardize the language among healthcare providers (4, 10), in addition to allowing the assessment of responses to therapies and data comparison over time.

Therefore, it is intuitive to think that the proper management of T1DM depends not only on the appropriate use of medications, but also on the patients’ knowledge about their treatment, healthy eating habits, exercise, and self-monitoring of blood glucose (11). Understanding the knowledge about diabetes in patients with T1DM can help to improve the quality of care and serve as a starting point for knowing how to involve the patients in their own care. Thus, healthcare providers ensure that patients receive the necessary support to understand, to assess, and to apply disease management guidelines to the process of managing their health. Thus study aimed to evaluate the association between knowledge about the disease, adherence to self-care, and glycemic control in people with T1DM.

## SUBJECTS AND METHODS

### Study design and setting

This is a descriptive cross-sectional study, guided by the Strengthening the Reporting of Observational Studies in Epidemiology (STROBE) guideline, which contains items that should be included in observational studies (12).

The study was carried out in a public tertiary university hospital. Around 395,826 outpatient consultations are performed each year at this hospital and, in 2021, more than 67,000 teleconsultations were conducted (13). Endocrinologists, nurses, social workers, and nutritionists work at the institution's endocrinology outpatient clinic, the research field.

### Population and sample

The population consisted of patients diagnosed with T1DM, with regular follow-ups at the institution's endocrinology outpatient clinic. All patients with T1DM treated at the institution's endocrinology outpatient clinic in the last two years were selected by a query request from keyworded electronic medical records. For inclusion in the study, participants had to be aged over 18 years and diagnosed with T1DM. Exclusion criteria were having a record of another type of diabetes (type 2 diabetes, maturity-onset diabetes of the young (MODY), latent autoimmune diabetes in adults (LADA), or an uncertain type of diabetes), cognitive impairment, pregnancy, death, and outpatient discharge.

To calculate the power of the sample, the online version of Power and Sample Size Health was used (14). Considering the 198 participants (Flowchart 1), 5% significance level, 0.3 Cohen's W effect size, and 1 degree of freedom as obtained by Borba and cols. (4), the power to test whether there is an association between knowledge and self-care in our study was 98.8%.

### Data collect

Data collection was carried out from January to March 2021, by telephone, due to social isolation measures implemented to reduce COVID-19 transmission. The calls were made by three researchers during business hours, that is, from 8 a.m. to 6 p.m. Patients were asked about their interest in participating in the survey by telephone and their availability to answer questions during the call, or if they wished to schedule it to another occasion. The questionnaires were answered by the participants during the phone calls, which were recorded and the participants were asked to answer, before the application of the questionnaires, if they agreed to participate in the research.

To facilitate the completion of the participants’ answers, an online form was created to collect data on the studied variables, including: medical record number, telephone, sex, age, schooling level, time of diagnosis, smoking status, value of the last glycated hemoglobin (HbA1c), comorbidities (cardiovascular diseases, dyslipidemia, arterial hypertension, diabetes kidney disease, neuropathy, foot injuries, previous amputations, and psychiatric conditions), Diabetes Knowledge Questionnaire (DKN-A), and Self-Care Inventory-Revised (SCI-R) validated for Brazilian Portuguese (15, 16). The DKN-A is a 15-item multiple-choice questionnaire on different aspects related to general knowledge of diabetes. Scale ranges from 0 to15 and each item is measured with a score of one (1) for correct answers and zero (0) for incorrect answers. Items one to 12 require a single correct answer. For items 13-15, some answers are correct, and all must be checked to obtain a score of one. A score greater than eight indicates knowledge about diabetes (15). Notably, in the results presentation, participants with scores from 0 to 8 were classified as “low knowledge” and above 9 as “satisfactory knowledge”.

The SCI-R has 14 items on a 5-point Likert scale (1 = never; 5 = always) that reflects how the participants followed the self-care recommendations during the last two months; higher scores indicate greater adherence, and the cut-off value to classify a patient as having a greater or lesser adherence score is 48 (16). In this case, when presenting the results, participants with scores below 48 were referred to as having lesser adherence to self-care and scores above 49 as having greater adherence.

To establish adequacy or lack of it for glycemic control, individualized goals were used. Participants with a history of ischemic heart disease, frequent episodes of hypoglycemia, severe visual impairment, those who underwent hemodialysis or peritoneal dialysis, and underwent only two or fewer capillary blood glucose tests per day were considered for a flexible target (HbA1c ≤ 8.0%). For all other participants, strict glycemic control was considered adequate (HbA1c target ≤ 7.0%). Patients who were within the glycemic target were considered to have good control and the others to be inadequate.

The primary outcome of the study was the presence of an association between diabetes knowledge and self-care. The secondary outcome included the presence of an association between diabetes knowledge and HbA1c levels.

The study included a pilot plan, to identify possible errors in the questionnaires and to reduce biases. The pilot plan was carried out with four patients with type 2 diabetes, not included in the study sample.

### Data analysis

Data analysis was performed using the Statistical Program Package for the Social Sciences (SPSS) version 22.0. Categorical variables were described by absolute number and percentile and continuous variables were described as mean and standard deviation in case of normal distribution; otherwise, data were described as median and interquartile range. Normality was defined by the Shapiro-Wilk test.

The analysis of the association between the results of the applied questionnaires (DKN-A and SCI-R) and glycemic control was performed using the chi-square test. To analyze the association between the DKN-A questionnaire and schooling level, the Mann-Whitney U test was performed for independent samples. Poisson's regression with adjustments for robust variances was used to identify significant predictors of knowledge about diabetes in relation to this variable being associated with self-care and schooling level. The statistical significance level was 5%.

### Ethical aspects

The study was approved by the Research Ethics Committee of the institution via Plataforma Brasil under CAAE number 20380919800005327, considering the prerogatives announced in Resolution 466/2012 of the Brazilian National Health Council. The researchers followed the institution's telephone call script for inviting participants to the research, which contained three options for the participant to choose to send an informed consent form (email, WhatsApp, or message), with the document being sent according to their preference. When handling the information, the researchers preserved the participants’ anonymity during the treatment and publication of the data.

## RESULTS

**Figure 1:**
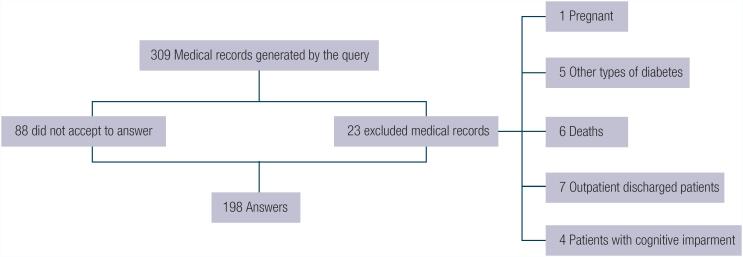
Patient inclusion flowchart

After the initial identification, 309 medical records were obtained from patients with T1DM aged 18 years or older, who had been treated at the institution in the last two years. Of these, 88 refused to answer the questionnaires and 23 patients were excluded (Figure 1).

In total, 198 patients answered the questionnaires; their mean age was 42 ± 12 years, 106 (53.5%) were women and 140 (70.8%) had satisfactory knowledge about diabetes. Greater self-care was observed in 65 patients (32.8%). Table 1 summarizes the other demographic and clinical characteristics and scores obtained by the participants in the questionnaires.

**Table 1:** Sociodemographic and clinical characteristics and scores attributed in the questionnaires of knowledge about diabetes (DKN-A) and self-care (SCI-R) of the study participants (n = 198)

Feature*		n = 198
Age (years)		42.9 ± 12.4
Sex		
	Female	106 (53.5)
	Male	92 (46.5)
Active smoking		21 (10.6)
Diagnosis time		22.5 ± 10.8
Glycated hemoglobin (%)		8.6 ± 1.6
	Adequate glycemic control	46 (23.2)
	Inadequate glycemic control	152 (76.8)
Comorbidities†		
	Systemic arterial hypertension	61 (30.8)
	Psychiatric illnesses	62 (31.3)
	Dyslipidemia	46 (23.2)
	Cardiovascular diseases	21 (10.6)
	Retinopathy	107 (54)
	Diabetes kidney disease	44 (22.2)
	Sensory neuropathy	42 (21.2)
	Foot injuries	16 (8.1)
Schooling level		
	Elementary school	78 (39.4)
	High school	78 (39.4)
	Higher education	42 (21.2)
Diabetes Knowledge Questionnaire (DKN-A)		9.5 ± 2.6
	Low knowledge	58 (29.2)
	Satisfactory knowledge	140 (70.8)
Self-care inventory revised (SCI-R)		44.6 ± 7.7
	Lesser self-care	133 (67.2)
	Greater self-care	65 (32.8)

* Continuous variables were described by mean and standard deviation and categorical variables by absolute number and percentile. Sensory neuropathy was considered present when recorded in the medical record by the attending physician. Satisfactory knowledge was considered 9 or more correct answers. Greater self-care was considered a score greater than or equal to 49.

† More than one response to this variable was computed.

The analysis of the association between knowledge about diabetes and self-care by the DKN-A and SCI-R questionnaires, respectively, showed that among the participants with greater knowledge about the disease (n = 140), 58 (41.4%) had greater adherence to self-care (p < 0.001). Table 2 shows the evaluated variables (glycemic control, sex, time since diagnosis, and schooling level) according to the knowledge about diabetes (DKN-A) of the 198 participants.

**Table 2:** Characteristics of patients according to their knowledge about diabetes (DKN-A) (n = 198)

Characteristic*		DKN-A Low knowledge	DKN-A Satisfactory knowledge	p-value
Sex				0.154†
	Female	26 (44.8)	80 (57.1)	
	Male	32 (55.2)	60 (42.9)	
Diagnosis time				0.366‡
	Less than 5 years	4 (6.9)	9 (6.4)	
	Between 6 and 15 years	11 (18.9)	39 (27.9)	
	Between 16 and 25 years old	19 (32.8)	31 (22.1)	
	Over 26 years	24 (41.4)	61 (43.6)	
Schooling level				0.003‡
	Elementary school	31 (53.5)	47 (33.5)	
	High school	21 (36.2)	57 (40.8)	
	Higher education	6 (10.3)	36 (25.7)	
Self-care inventory revised (SCI-R)				<0.001†
	Lesser self-care	51 (87.9)	82 (58.6)	
	Greater self-care	7 (12.1)	58 (41.4)	
Glycemic Control				0.705†
	Adequate glycemic control	15 (25.9)	31 (22.2)	
	Inadequate glycemic control	43 (74.1)	109 (77.8)	

* Variables were described by absolute number and percentage. Satisfactory knowledge was considered 9 or more correct answers. Greater self-care considered a score greater than or equal to 49.

† Yates continuity correction test.

‡ Pearson's chi-square test. DKN-A: Diabetes Knowledge Questionnaire.

By the Poisson's regression model with adjustments for robust variances of the knowledge questionnaire for the self-care and schooling inventory, prevalence of satisfactory knowledge in participants with better self-care was 44.7% higher than the prevalence in those who showed lower adherence to self-care (odds ratio: 1.447, confidence interval: 1.235-1.696, p < 0.001). The prevalence of satisfactory knowledge about the disease among those with higher education was 42.2% higher than the prevalence among those with elementary school (odds ratio: 1.422, confidence interval: 1.143-1.770, p = 0.006).

Table 3 shows the evaluated variables (glycemic control, sex, time since diagnosis, and schooling level) according to the patients’ self-care (SCI-R). Among the 65 participants with higher self-care score on the SCI-R, 18 (27.7%) had adequate glycemic control (p = 0.390).

**Table 3:** Characteristics of patients according to adherence to self-care (SCI-R) (n = 198)

Characteristic*		SCI-R Lesser self-care	SCI-R Greater self-care	p-value
Sex				0.928†
	Female	72 (54.1)	34 (52.3)	
	Male	61 (45.9)	31 (47.7)	
Schooling level				0.002‡
	Elementary School	60 (45.1)	18 (27.7)	
	High school	53 (39.8)	25 (38.5)	
	Higher education	20 (15)	22 (33.8)	
Diagnosis time				0.823‡
	Less than 5 years	8 (6)	5 (7.7)	
	Between 6 and 15 years	33 (24.8)	17 (26.2)	
	Between 16 and 25 years old	35 (26.3)	15 (23.1)	
	Over 26 years	57 (42.9)	28 (43.1)	
Glycemic Control				0.390†
	Adequate glycemic control	28 (21.1)	18 (27.7)	
	Inadequate glycemic control	105 (78.9)	47 (72.3)	

* Variables described by absolute number and percentage; Greater self-care was considered a score greater than or equal to 49; SCI-R: Self-care inventory revised (SCI-R);

† Yates continuity correction test;

‡ Mann-Whitney U test.

## DISCUSSION

This study aimed to evaluate the association between knowledge about the disease, adherence to self-care, and glycemic control in people with T1DM. The results showed that participants with satisfactory knowledge about the disease present greater adherence to self-care in relation to people with low knowledge. However, glycemic control was not influenced by the participants’ level of knowledge: both patients with satisfactory and unsatisfactory knowledge about the disease had inadequate glycemic control.

In our sample of patients with T1DM, the average of knowledge about the disease was considered good, with most participants scoring more than nine correct answers in the DKN-A questionnaire, different from what was verified in an Indian study, in which the score of knowledge about the disease was medium (7) and in an Ethiopian study, in which the level of knowledge was low (8). Furthermore, in our study, having knowledge about the disease greatly influenced adherence to self-care, a behavior that is idealized for all patients with chronic diseases (17). Importantly, knowledge about diabetes encompasses the basic physiology of the disease, management of hypoglycemia, food groups and their substitutions, management of diabetes in intercurrences, and the general principles of care for the disease (4). Self-care is related to a brief and psychometric measure of perceptions of adherence to recommended self-care behaviors in patients with diabetes (16). Following the self-care guidelines provided by the health team is essential for adhering to the treatment, however, patients needs to be active and show care attitudes towards their disease.

Our study showed that satisfactory knowledge about diabetes in respondents with higher education was greater in relation to participants with lesser knowledge about the disease. In addition, greater self-care was presented in patients with higher education in relation to participants with lesser self-care. We also observed that satisfactory knowledge was more prevalent among women and this patient profile was also observed in people with type 2 diabetes (2,11,18). Better knowledge scores were also associated with a higher schooling level in people with T1DM in India, Ethiopia, and Canada (7,8,19). People with a low schooling level tend not to value preventive actions, underestimating the severity of the disease and postponing the search for assistance, which impairs commitment to their treatment (9). Although knowledge of the disease alone does not guarantee the necessary changes in behavior, assessing the patients’ knowledge of the disease is essential for designing educational health interventions (11).

Regarding glycemic control, the results are worrying, as most patients with inadequate control showed satisfactory knowledge about the disease. Knowledge and awareness of diabetes regarding the biology of the pathology, its ongoing health implications, and how to manage the condition are vital to understand the need to maintain good glycemic control (20). However, based on the results, many people diagnosed with diabetes, even with good scores on the knowledge questionnaire, may not have a clear understanding of disease control goals or how to effectively manage their health. We understood that either the questionnaire is not sensitive enough to capture the entire knowledge that the patient has about all aspects of diabetes, or, more than having knowledge, other domains of these patients’ attitudes need to be activated so that there is an effect on attitude changes that lead to better glycemic control.

As for the time of diagnosis, the results show that knowledge about diabetes was not associated with the duration of the disease, unlike what was reported in a study carried out in Ethiopia with the same population (8). This data suggests a reflection on the effective communication of diabetes education to patients with T1DM. The strengthening of information, education, and effective communication on diabetes is of paramount importance (8). Adherence to self-care also showed no association with the duration of the disease. It is expected that the longer the duration of the disease, the more knowledge about diabetes and its treatment the patients should have (4). However, we did not observe this trend in our study nor in another study carried out in primary care in Northeastern Brazil (4), in which the negative attitude towards self-care also showed no difference between different durations of the disease. Notably, age and duration of diabetes are known but not modifiable risk factors for microvascular and cardiovascular outcomes and mortality in T1DM patients (21).

In our study, adherence to self-care practice in diabetes was lower in patients who had inadequate glycemic control compared to participants with greater adherence to self-care. A Brazilian multicenter study showed that inadequate glycemic control, common in Brazilians with T1DM, is associated with lower schooling level, insufficient self-perception of adherence and inadequate monitoring of glycated hemoglobin levels (22). A study carried out with the same population evaluated the practice of foot care, demonstrating a disagreement with the knowledge presented by the participants. Among the interviewees, 32.7% had good knowledge about foot care, but only 12.2% practiced it (23). However, in patients with type 2 diabetes from another population, better self-care practices were associated with greater knowledge about diabetes and lower levels of HbA1c (11). Better self-care was also associated with a 0.4 fold increase in the quality of life of participants with type 2 diabetes in another study (24). However, it is known that in Brazil, despite the free supply of insulin and supplies for self-care (needles, glucometers, reagent strips), the patient cannot always access these supplies, because of lack of knowledge or because of differences in providing this support between different municipalities and states (25, 26). These circumstances can affect adherence to self-care with the disease. Furthermore, in regions with a lack of medical resources, individuals with T1DM tend to die early from acute metabolic complications or infections (27).

Another factor to consider, in relation to the self-care result presented by the participants, is the date of data collection, which took place during one of the lockdowns imposed by the coronavirus pandemic. The COVID-19 outbreak has caused many municipalities worldwide to have their routines completely changed by social isolation measures, suddenly changing the daily routine of people with diabetes, increasing sedentary behavior and changing dietary patterns (28–30). These circumstances imposed by the pandemic resulted in changes in self-care and glycemic control, especially in patients on complex therapeutic regimens, such as people with diabetes (30, 31).

This study has some limitations, such as its cross-sectional design and the population belonging to only one tertiary care center, but a reference in this type of care and with patients from all over the state. Therefore, the characteristics of the participants can be considered representative of the population served by public hospitals and the results should be explored considering cultural and economic aspects, which affect the disease management. Another limitation includes the possibility of self-report bias, as patients may not be willing to reveal deficiencies in self-care knowledge and practices and may not be accurate all the time.

In any case, our results draw the attention of nurses, physicians, and other healthcare providers to the challenges of improving self-care and, consequently, health among T1DM patients. In addition, our study makes a strong case for diabetes educators to actively involve their patients in a more participatory position, in order to put their knowledge about the disease into practice, so that the necessary care with diabetes is carried out, thus reducing complications that health carelessness can bring.

In conclusion, knowledge about the disease was associated with greater adherence to self-care in people with T1DM, but this was not reflected in better glycemic control. Improvement in self-care, however, can be reflected in other health domains of the person with diabetes, therefore, this result should be valued. Patients should be encouraged to introduce their knowledge about the disease into their routine and to improve self-care at each follow-up appointment with healthcare providers. In addition, it is essential to seek alternatives to strengthen the information provided to these patients, which may reflect on better glycemic control. Thus, this study contributes to the field of nursing by bringing a relevant analysis of the knowledge about the disease of adult patients with T1DM and draws attention to the urgent need to search for new tools that can improve education in relation to self-care and glycemic control.
